# Metabolic reprogramming in osteosarcoma

**DOI:** 10.1002/pdi3.18

**Published:** 2023-07-26

**Authors:** Yulu Shi, Xiaohan Yue, Qing Luo

**Affiliations:** ^1^ Stem Cell Biology and Therapy Laboratory The Children's Hospital of Chongqing Medical University National Clinical Research Center for Child Health and Disorders Ministry of Education Key Laboratory of Child Development and Disorders Chongqing Key Laboratory of Pediatrics Chongqing China

**Keywords:** amino acid, fatty acid, glycolytic, metabolism, osteosarcoma, OXPHOS

## Abstract

Tumor cells undergo metabolic reprogramming to meet their energy and anabolic demands to maintain their malignant phenotype. Activation of oncogenes and deletion of tumor suppressors promotes metabolic reprogramming in cancer by directly or indirectly regulating enzymatic activities associated with metabolic pathways. Metabolic reprogramming in tumor cells mainly involves the glycolytic pathway, pentose phosphate pathway, serine synthesis pathway, enhanced glutamine metabolism or fatty acid anabolism, and abnormal mitochondrial oxidative phosphorylation (OXPHOS). The tricarboxylic acid (TCA) cycle is the central pathway of mitochondrial OXPHOS, and glucose, amino acid and fatty acid metabolism are associated with the TCA cycle. Metabolic abnormalities and rewiring of metabolic pathways are also present in Osteosarcoma (OS). The abnormal metabolic pattern in OS is associated with cell proliferation, migration, invasion and drug resistance. This review summarizes the current studies on glycolysis, amino acid metabolism, lipid synthesis and the TCA cycle related to OS.

## 1 BACKGROUND

Osteosarcoma (OS) is children's and adolescents' most common malignant bone tumor. Osteosarcoma occurs in the location of rapid bone growth (epiphysis of long bones). The primary treatment modality for OS is neoadjuvant chemotherapy combined with limb‐preserving surgery. The main chemotherapeutic agents used are cisplatin (CIS), methotrexate (MTX), doxorubicin (DOX), etoposide (ETOP), and isocyclophosphamide.^1^ Multidrug chemotherapy regimens have increased long‐term survival to 65%–70% in patients with limited OS.^2^ However, patients with OS are prone to chemoresistant lung metastases, leaving event‐free survival outcomes below 30%.^3^ Metabolite screening has identified progression‐dependent metabolic alterations in OS,^4^ which meets cancer cells' rapid growth and proliferation needs.

**TABLE 1:** Glycolytic related genes in Osteosarcoma (OS).

Gene	Pathway	Target gene	Effect
miR‐328‐3p^16^		GLUT1	Object
miR‐21‐5p^21^	Wnt/*β*‐catenin	GLUT1	Support
lncR‐HAND2‐AS1^17^	HIF1α	GLUT1	Object
Osteopontin^22^		GLUT1	Support
LAIR‐1^19^		GLUT1	Object
p53^20^		GLUT1	Object
miR‐21‐5p^21^	Wnt/*β*‐catenin	HK2	Support
lncR‐HAND2‐AS1^17^, ^18^	HIF1α	HK2	Object
LncR‐PVT1^35^	miR‐497/HK2	HK2	Support
LncR‐TUG1^33^		HK2	Support
LncR‐SARCC^38^	miR‐143	HK2	Object
Circ‐CTNNB1^32^	RBM15	HK2	Support
CircFAT1 (e2)^36^	miR‐181b	HK2	Support
miR‐615^37^	PI3K/AKT	HK2	Object
ROCK2^34^	PI3K/AKT	HK2	Support
LncR‐XLOC‐005950^40^	hsa‐miR‐542‐3p	PFKM	Support
miR‐26b^42^		PFKFB3	Object
LncR‐KCNQ1OT1^43^	miR‐34c‐5p	ALDOA	Support
S1PR3^47^	YAP/c‐MYC/PGAM1	PGAM1	Support
miR‐21‐5p^21^	Wnt/*β*‐catenin	PKM2	Support
LncR‐CCAT1^52^		PKM2	Support
miR‐491‐5p^53^		PKM2	Object
IRF7^54^		PKM2	Object
TRIM58^55^		PKM2	Object
miR‐21‐5p^21^	Wnt/*β*‐catenin	LDHA	Support
CircCNST^62^	miR‐578‐LDHA/PDK1	LDHA	Support
CircATRNL1^63^	miR‐409‐3p/LDHA	LDHA	Support
miR‐323a‐3p^64^		LDHA	Object
miR‐329‐3p^65^		LDHA	Object
KDM6B^61^		LDHA	Support
miR‐186^27^		HIF‐1ɑ	Object
TGF‐β^29^	SDH/HIF1α	HIF‐1ɑ	Support
KRT17^28^	AKT/mTOR/HIF1α	HIF‐1ɑ	Support
CircECE1^24^		c‐Myc	Support
Her4^25^		c‐Myc	Support
PDGFRβ^26^	PI3K/AKT/mTOR	c‐Myc	Support

Abbreviations: ALDOA, Aldolase; GLUT1, glucose transporter type 1; HIF‐1ɑ, hypoxia inducible factor 1 subunit alpha; HK2, hexokinase 2; LDHA, Lactate dehydrogenase A; PFKFB3, 6‐phosphofructo‐2‐kinase/fructose‐2,6‐bisphosphatase 3; PFKM, phosphofructokinase skeletal muscle; PGAM1, phosphoglycerate Mutase 1; PKM2, pyruvate kinase M2.

Warburg first proposed that cancer cells rely on aerobic glycolysis rather than oxygen‐consuming mitochondrial metabolism, even sufficient oxygen. Glycolysis in cancer cells has been well studied (Table 1), and it is believed that this mode of metabolism is low in productivity but fast in efficiency. Cancer cells, in turn, promote a higher rate of glycolysis by increasing glucose uptake.^5^ Increased glycolysis is also found to contribute to the biosynthesis of amino acids, nucleotides and lipids. Glycolytic intermediates provide the necessary anabolism for cell proliferation and tumor growth. The Warburg effect reconnects amino acid and lipid metabolism. As a result, other metabolic modalities have received increasing attention. The pentose phosphate pathway (PPP) helps glycolytic cancer cells to meet their nucleic acid and fatty acid synthesis requirements and to survive under stressful conditions. Cancer cells selectively take up exogenous serine or synthesize serine via the serine synthesis pathway (SSP), which is converted into intracellular glycine and one‐carbon units for nucleotide biosynthesis.^6^ The balance of folate metabolism and redox homeostasis necessary for cancer cell proliferation may require active serine synthesis.^7^ Glutamine metabolism drives cancer growth and progression for DNA damage repair, epigenetic modification and immune regulation.^8^ Glutamine fuels the tricarboxylic acid (TCA) cycle and acts as a redox donor during the Warburg effect.^9^ Increased fatty acid synthesis in cancer cells facilitates membrane biosynthesis and signaling molecules. Glucose metabolism, amino acid metabolism and lipid metabolism intersect in the TCA cycle. Genetic defects and oncogenic signaling can affect TCA cycle activity.^10^ The TCA cycle intermediates can influence cancer development by regulating cellular metabolism and signaling.^11^ Given that metabolic reprogramming is increasingly studied in OS and plays an essential role in the development of OS, a review of the metabolic alterations involved in OS and their impact on the development of OS is presented.

## 2 GLYCOLYSIS IN OSTEOSARCOMA

### 2.1 Glucose uptake

Glucose serves as the body's energy supply, and glucose uptake increases in cancer cells compared to normal cells. The enhanced glucose uptake by cancer cells is the basis for positron emission tomography with 18‐fluorodeoxyglucose (FDG) and is used in clinical applications. Glucose is hydrophilic, while the main body of the cell membrane is hydrophobic, so glucose transport requires the aid of glucose transport proteins. Increased glucose uptake by cancer cells is due to the overexpression of glucose transporter proteins, particularly GLUT1 and GLUT3, which are responsive to hypoxia, and other glucose transporter proteins.^12^ GLUT1 is expressed overly in OS tissues, and patients with high GLUT1 expression have lower median survival than those with the standard term.^13^ GLUT1 expression correlates negatively with microvessel density values in OS.^14^ Downregulation of GLUT1 inhibits glucose uptake, growth and invasion of OS cells.^15^ Some studie have found that non‐coding RNAs: miR‐328‐3p and lncR‐HAND2‐AS1, and protein‐coding genes: LAIR‐1 and p53 can inhibit GLUT1 expression to reduce glycolysis and have an effect on cell proliferation or epithelial‐mesenchymal transition. In contrast, inhibition of the above genes has leaded to increased glucose uptake and cellular energy supply.^16^, ^17^, ^18^, ^19^, ^20^ The miR‐21‐5p and Osteopontin have been upregulated in OS, which can increase GLUT1 expression to enhance cellular glucose uptake, and increase the Warburg effect.^21^, ^22^ LncR‐HAND2‐AS1 and Osteopontin can also affect GLUT3 expression,^18^, ^22^ and the tumor suppressor p53 can also affect GLUT4 expression.^20^ Activation of oncogenes and deletion of tumor suppressors promotes metabolic reprogramming in cancer. GLUT1 can be activated by oncogenes cMyc and HIF‐1ɑ.^23^ Circ‐ECE1, Her4, and PDGFRβ increase glucose uptake and promote metabolic reprogramming in OS through cMyc.^24^, ^25^, ^26^ MiR‐186, KRT17 and TGF‐β promote glucose uptake and glycolysis in OS cells via HIF‐1ɑ.^27^, ^28^, ^29^ The miR‐200c plays a central role in glucose metabolism in cancer cells, and CircCSPP1 may influence miR‐200c maturation to regulate glucose uptake and cell proliferation to promote OS.^30^ By interfering with glucose uptake, glycolysis is influenced at the source, affecting OS cells' energy supply. The increased glucose consumption of cancer cells compared to normal cells will facilitate the provision of the carbon source required to maintain the cancer cells' growth and rapid proliferation.

### 2.2 The first key step of glycolysis

Hexokinase (HK) provides the primary role for the cellular utilization of glucose, and HK2 is a major player in maintaining a highly malignant state, with HK2 catalyzing the first irreversible step of glycolysis, converting glucose to glucose‐6‐phosphate. HK2 is overexpressed in approximately 80% of OS specimens, and silencing of HK2 reduces aerobic glycolysis and cell proliferation and increases apoptosis.^31^ Several studies have found that non‐coding RNAs: miR‐21‐5p, Circ‐CTNNB1 and LncR‐TUG1, and ROCK2 promote the glycolytic process and activate OS progression through HK2 expression and that inhibition of the above genes reduces glucose consumption lactate production and OS cell viability.^21^, ^32^, ^33^, ^34^ Han et al. found that LncR‐PVT1 promoted glycolysis and tumor progression by regulating the miR‐497/HK2 axis in OS.^35^ Gu et al. found that CircFAT1(e2) promoted glycolysis and tumor progression by miR‐181b regulates HK2 expression to promote OS progression and metastasis.^36^ Other studies have found that non‐coding RNAs: LncR‐HAND2‐AS1 and miR‐615 inhibit HK2 to exert tumor‐suppressive effects in OS.^18^, ^37^ Wen and his colleagues found that LncR‐SARCC sensitized OS to cisplatin by inhibiting HK2 expression through miR‐143.^38^

### 2.3 The second key step of glycolysis

Fructose‐6‐phosphate 1‐kinase (PFK‐1) catalyzes the second irreversible step of glycolysis, converting fructose‐6‐phosphate to fructose‐1,6‐bisphosphate. Glycolytic phosphofructokinase skeletal muscle (PFKM), a skeletal muscle isoform, is an essential mediator of cellular metabolism.^39^ PFKM is expressed highly in OS cells and tissues, and reducing PFKM expression by knocking down lncRNA XLOC_005950 can inhibit glycolysis and proliferation of OS cells.^40^ Fructose 2,6‐bisphosphate (F2,6P2) is a potent activator of PFK‐1, and 6‐phosphofructo‐2‐kinase/fructose‐2,6‐bisphosphatase 3 (PFKFB3) is an essential regulator of glycolysis that is closely related to tumorigenesis and development.^41^ Du and his colleagues found that MiR‐26b suppressed malignant behavior and induced apoptosis by downregulating PFKFB3‐driven glycolysis in OS cells.^42^

### 2.4 Intermediate steps of glycolysis

Aldolase (ALDOA) is an enzyme that catalyzes reversible reactions during glycolysis and plays a role in glycolysis and gluconeogenesis. Aldolase, upregulated in OS cells and tissues, plays a crucial role in OS progression and metastasis and is associated with poorer overall survival.^43^, ^44^, ^45^ Long et al. found that the knockdown of ALDOA suppressed MMP‐2 expression in OS cells and reduced invasive capacity and survival.^44^ Shen and his colleagues found that LncR‐KCNQ1OT1 played an essential role in glucose metabolic reprogramming by competitively binding miR‐34c‐5p to promote ALDOA expression in OS.^43^ Phosphoglycerate Mutase 1 (PGAM1) is another glycolytic enzyme catalyzing the interconversion between 2‐phosphoglycerate and 3‐phosphoglycerate. Phosphoglycerate Mutase 1 is overexpressed in many cancers and promotes their progression.^46^ A study found that the S1P/S1PR3 axis initiated the Warburg effect by activating the YAP/c‐MYC/PGAM1 pathway and that the S1PR3 antagonist TY52156 exhibited synergistic inhibition of OS cell growth with methotrexate in vitro and in vivo.^47^

### 2.5 The last key step of glycolysis

The dimer pyruvate kinase M2 (PKM2) catalyzes the conversion of phosphoenolpyruvate to pyruvate in the last irreversible step of glycolysis. Pyruvate kinase M2 is abundantly expressed in most tumor cells, shifting glucose metabolism from the standard respiratory chain to lactate production^48^ and is involved in cell proliferation through metabolic reprogramming.^49^ Liu and Yuan et al. found that the overexpression of PKM2 predicted poor prognosis in OS patients,^50^ and knockdown of PKM2 inhibited OS cell proliferation, invasion, and migration, as well as induced apoptosis in vitro, and slowed tumor growth in vivo.^51^ Some studies have found that miR‐21‐5p and LncR‐CCAT1 increase the Warburg effect by increasing PKM2 expression to promote glycolysis, which promotes OS tumorigenesis.^52^ Other studies have found that miR‐491‐5p, IRF7 and TRIM58 inhibit tumor progression in OS cells by suppressing PKM2 activity.^53^, ^54^, ^55^ Shang et al. found that Metformin increased the sensitivity of OS stem cells to cisplatin by inhibiting the expression of PKM2.^56^

### 2.6 Lactic acid production

Lactate dehydrogenase A (LDHA) catalyzes the last step of the aerobic glycolytic pathway converting pyruvate to lactate. Lactate dehydrogenase A is aberrantly highly expressed in many malignancies and promotes the malignant progression of tumors by increasing glycolytic regulation of many cancer‐related proteins.^57^ LDHA is typically upregulated in OS cell lines.^58^ Reducing LDHA activity with inhibitors of LDHA or shRNA leads to lower lactate levels in the culture medium of OS cells. It reduces OS cells' proliferation and invasive capacity by inhibiting the Warburg effect.^58^, ^59^, ^60^ A recent study has found that KDM6B promotes OS cell metastasis by regulating LDHA expression.^61^ The knockdown of Circ‐CNST can antagonize the malignant behavior and glycolysis of OS cells by regulating the miR‐578‐LDHA/PDK1 axis.^62^ Zhang et al. found that CircATRNL1 enhanced glycolysis via the circATRNL1/miR‐409‐3p/LDHA axis and promoted OS progression.^63^ Other studies have found that non‐coding RNAs: miR‐323a‐3p and miR‐329‐3p inhibit OS cell proliferation by targeting LDHA to repress OS glycolysis.^64^, ^65^

### 2.7 Glycolytic branch metabolism

The PPP branches off from the first key step of glycolysis, producing ribulose‐5‐phosphate for purine nucleotide synthesis and Nicotinamide adenine dinucleotide phosphate (NADPH) for reactive oxygen species (ROS) scavenging and fatty acid synthesis. The glucose‐6‐phosphate dehydrogenase enzyme (G6PD) catalyzes the first irreversible reaction in the oxidative branch of the PPP. Glucose‐6‐phosphate dehydrogenase enzyme determines the flux of glucose‐6‐phosphate (G‐6‐P) directly into the PPP pathway and catalyzes the G‐6‐P conversion to gluconolactone 6‐phosphate, accompanied by the production of the reductant NADPH. Upregulation of G6PD is present in various tumors, and increased G6PD expression leads to increased biosynthesis of nucleotides and lipids necessary for cell division and proliferation, favoring tumor cell proliferation.^66^, ^67^ One study found that approximately 70% of OS samples showed high G6PD expression.^68^ Wang et al. found that LncR‐OR3A4 affected the proliferation of OS cells by regulating the miR‐1207‐5p/G6PD axis.^69^ The PPP is a major source of NADPH and ribulose phosphate, providing a carbon source and hydrogen donor for various metabolisms in the body. The PPP plays a key role in helping glycolytic cancer cells meet their nucleotide synthesis and combat oxidative stress. Current studies on the PPP in OS are limited. Another branch of glycolysis is the SSP, where the glycolytic intermediate 3‐phosphoglycerate (3PG) is converted to serine by a three‐step enzymatic reaction elaborated in OS amino acid metabolism.

## 3 AMINO ACID METABOLISM IN OSTEOSARCOMA

### 3.1 Serine synthesis pathway

Amino acids are involved in biosynthesis and energy supply and help maintain redox homeostasis. The SSP is sarcoma's most widely studied amino acid biosynthetic pathway.^70^ Downregulation of the SSP inhibited the malignant behavior of OS cells.^71^ The 3‐phosphoglycerate dehydrogenase (PHGDH) is the first rate‐limiting enzyme in the SSP. Abnormally high expression of PHGDH is present in OS clinical samples and cells.^72^ One study found that inhibition of mTORC1 reduced serine and glycine in OS cells by regulating glycolysis and serine/glycine synthesis gene expression.^73^ Not only do serine and glycine provide precursors for nucleic acid, protein and lipid synthesis for cancer cell growth, but they also increase the antioxidant capacity of tumor cells. Wang and his colleagues found that the mTORC1/serine/glycine metabolic axis promotes OS proliferation and antioxidant capacity in response to environmental stress, ultimately leading to cell survival.^73^ Another study found that inhibition of PHGDH in OS cell lines reduced cell proliferation but did not lead to cell death; inhibition of PHGDH led to the accumulation of branched‐chain amino acids, methionine cycle intermediates and unsaturated fatty acids in OS cells, resulting in mTORC1 activation.^72^ Pruksakorn et al. found that serine‐threonine kinase receptor‐associated protein was up‐regulated during OS progression, enhancing the metastatic ability of OS cells.^74^ Some studies have found that inhibition of serine/threonine protein kinases: glycogen synthase kinase‐3β (GSK‐3β), cyclin G‐associated kinase, Polo‐like kinase 1, glycogen synthase kinase 3β (GSK3β), and Polo‐like kinase 2 reduce the malignant phenotype of OS.^9^, ^75^, ^76^, ^77^, ^78^, ^79^ Other studies have found that inhibition of serine/threonine protein phosphatases: serine/threonine protein phosphatase 2A (PP2A) and serine/threonine phosphatase type 5 reduce the viability of OS cells.^80^, ^81^, ^82^

### 3.2 Glutamine metabolism

Glutamine metabolism is critical for normal or tumor cell proliferation, differentiation status, stress recovery, and survival.^83^ Increased glutamine uptake, consumption and Glutaminase (GLS) dysregulation are hallmarks of many types of cancer. Glutamine synthetase catalyzes the conversion of glutamate and ammonia to glutamine. Cellular glutamine uptake is via amino acid transporter proteins. Glutaminase catalyzes the conversion of glutamine to glutamate. GS and GLS are therapeutic targets for cancer.^84^ The growth of OS cells depends on glutamine.^68^, ^85^, ^86^, ^87^ Anso and his colleagues found that MYC‐induced glutamine metabolism was essential for MYC‐dependent proliferation and survival in OS cells.^86^ The amino acid transporter protein ASCT2 is considered the major glutamine transporter protein, and ASCT2 is expressed overly in various cancers and is strongly associated with poor prognosis.^88^ However, Bror et al. Found that 143B OS cells did not require ASCT2 to maintain rapid cell growth, and 143B OS cells maintained glutamine metabolism through amino acid transporter proteins: SNAT1 and SNAT2.^85^, ^89^ In Tardito's study, inhibition of Glutamine synthetase activity enhanced the antiproliferative and cytotoxic effects of L‐asparaginase.^90^ GLS1 is expressed highly in OS, and the higher the expression, the lower the survival rate of patients.^91^ Inhibition of GLS1 limits the growth and metastasis of OS cells. Ren et al. discovered that the OS cells with GLS1 inhibitor (CB‐839) in combination with metformin showed an overall decrease in glycolysis and TCA cycle function, as well as an increase in fatty acid oxidation (FAO) and pyrimidine catabolism.^92^ In zhou's research, miR‐141‐3p promoted cisplatin sensitivity by targeting GLS to regulate glutamine metabolism.^93^

## 4 LIPID SYNTHESIS IN OSTEOSARCOMA

### 4.1 De novo lipid synthesis

While normal cells rely mainly on exogenous fatty acid uptake, tumor cells increase de novo lipid synthesis. This alteration facilitates the synthesis of lipid membranes and signaling molecules in tumor cells. Lipid synthesis includes the fatty acid synthesis pathway as well as the mevalonate pathway. The ATP‐citrate lyase (ACLY) converts mitochondria‐derived citrate to acetyl coenzyme A, a precursor of the fatty acid synthesis pathway and the mevalonate pathway (MVA).^94^ One study found that miR‐22 reduced de novo lipid synthesis by inhibiting the expression of ACLY helped hinder OS cell proliferation and invasion.^95^ Another study found that LncRNA XIST from BMSC‐derived exosomes could enter OS cells, bind and down‐regulate miR‐655 levels, leading to elevated ACLY levels, thereby increasing lipid synthesis and *β*‐linked protein signaling activity and promoting malignant behavior in OS.^96^ Specific studies on ACLY in OS are lacking, but from the current study, it appears that increased lipid synthesis due to increased ACLY levels is also detrimental to OS.

### 4.2 Fatty acid synthesis pathway

Fatty acid synthase (FASN) is a key enzyme that catalyzes the terminal step of the fatty acid synthesis pathway. Tumor‐associated FASN confers survival advantage rather than acts as an energy store in anabolism.^97^ FASN is involved in pulmonary metastasis of OS,^98^ and direct inhibition of FASN expression by inhibitors or siRNA reduced the growth and metastasis.^99^, ^100^ Several studies have found that miRNA‐424, miRNA‐195, *α*‐linolenic acid, and lapatinib can inhibit FASN to reduce the malignant phenotype of OS.^101^, ^102^, ^103^, ^104^ Some studies have found that FASN inhibition inhibited OS metastasis by downregulating the HER2/PI3K/AKT signaling pathway.^105^, ^106^ Yet other studies found by PI3K/AKT signaling pathway to affect FASN expression.^104^, ^107^

### 4.3 The mevalonate pathway

Metabolites of the MVA pathway are essential for cancer cell survival and growth.^108^, ^109^ HMG‐CoA reductase (HMGCR) catalyzes the production of MVA from 3‐hydroxy‐3‐methylglutaryl coenzyme A. HMG‐CoA reductase expresses highly in OS cells, and statin‐induced inhibition of HMGCR reduces cell migration.^110^, ^111^, ^112^, ^113^, ^114^ Lovastatin (Lov), bisphosphonates (BP) and metformin (Met) can interfere with the mevalonate pathway (MP) and inhibit the progression of OS or affect the efficacy of anticancer drugs and DNA damage response.^115^, ^116^, ^117^, ^118^, ^119^

## 5 THE TRICARBOXYLIC ACID CYCLE IN OSTEOSARCOMA MITOCHONDRIA

### 5.1 Mitochondria in osteosarcoma

Mitochondrial oxidative phosphorylation (OXPHOS) is the hub that connects nutrient metabolism. Two different metabolic patterns of OS cells are identified: one is a highly differentiated, actively respiring cell line (Saos2 and HOS); the other is a highly malignant, poorly respiring and highly glycolytic cell line (LM7 and 143B).^120^, ^121^ OS showing the Warburg effect has a large number of swollen mitochondria and the presence of mitochondrial dysfunction, which was associated with mitochondrial permeability transition or an abnormal increase in mitochondrial replication (overexpression of mitochondrial single‐stranded DNA‐binding proteins).^120^, ^121^ The OS has a high frequency of mutations throughout the mtDNA, some of which are functionally relevant mutations with potentially detrimental effects on the mitochondrial OXPHOS system.^122^ In OS tissues, the average mtDNA number is reduced significantly, mutations in the D‐loop region of mitochondrial DNA occur frequently, and the mtDNA number is decreased approximately 2‐fold in metastatic tumors compared to non‐metastatic low‐grade tumors.^123^, ^124^ Different mtDNA mutations alter tumor progression according to the degree of respiratory chain complex I (CI) damage.^125^ CI is the first component of mitochondrial OXPHOS. It controls energy metabolism and maintains cellular redox status by regulating NAD+/NADH ratio, and ROS production has a crucial role.^126^ Tumor‐promoting mtDNA mutations increase cell proliferation by inducing the Warburg effect, increasing oxidative stress levels and activating the PI3K/Akt pathway. Antitumorigenic mtDNA mutations reduce tumorigenic potential by impeding hypoxia adaptation capacity and energy depletion.^125^, ^127^

### 5.2 Succinate dehydrogenase

Succinate dehydrogenase (SDH), also known as complex II, catalyzes the oxidation of succinate to fumarate in the TCA cycle and reduces ubiquinone to ubiquinol in the electron transport chain (ETC).^11^ Xu et al. found that reduced SDH expression in OS cells led to an accumulation of succinate, and TGF‐β reduced SDH expression by decreasing STAT1, leading to upregulation of HIF1α, which alters glucose metabolism and exacerbates chemoresistance in OS cells.^29^ Ziemann and colleagues discovered that MiR‐101‐3p was a tumor suppressor that regulates mitochondrial metabolism of OS cells by regulating mtDNA transcription and complex II.^128^

### 5.3 Isocitrate dehydrogenase

Isocitrate dehydrogenases (IDH1 and IDH2) catalyze the second irreversible step of the TCA cycle to decarboxylate isocitrate and NADP + to *α*‐ketoglutarate and NADPH. Α‐ketoglutarate inhibits the proliferation of OS cell lines in a concentration‐dependent manner.^129^ IDH1 and IDH2 mutations have been identified in various myeloid malignancies and solid tumors.^130^ IDH1/2 mutations also exist in OS.^131^, ^132^ Several studies have found that IDH1 and IDH2 negatively correlate with the pathological grade and metastatic potential of human OS, with upregulation of IDH1 suppressing the malignant biological behavior of OS cells and promoting apoptosis and downregulation of IDH2 exacerbating malignant progression by increasing NF‐KB and MMP‐9 activity.^133^, ^134^ Hu et al. found that OS patients with high expression of IDH1 had very high p53 levels.^135^ Liu et al. discovered that the HIF‐1α negatively correlated with IDH‐1 and inhibited IDH‐1, thereby increasing the incidence of OS.^136^ However, Zheng and his colleagues found that high IDH1 levels were associated with poor prognosis in OS patients and that AHA1 promoted OS growth and metastasis by upregulating IDH1 and metabolic activity.^137^ Some tumors have mutations in the TCA cycle or ETC that disable mitochondrial OXPHOS, and tumor cells with defective mitochondria use reduced glutamine metabolism as the primary pathway for citrate formation. In the study of Zheng et al., the reduced glutamine‐dependent pathway was the main pathway for fatty acid production during cell growth in 143Bcytb (an OS cell with defective OXPHOS), and reduced carboxylation in 143Bcytb cells needs IDH1 and IDH2.^138^

## 6 SUMMARY AND OUTLOOK

In recent years, exploring abnormal metabolic patterns has received increasing attention in OS. The current study found increased glycolysis, serine biosynthesis, glutamine metabolism and lipid synthesis in OS cells. Enhanced aerobic glycolysis is associated with PI3K/AKT/mTOR pathway, oncogenes, tumor suppressors or non‐coding RNAs affecting the activity of glycolysis‐related enzymes: HK2, PFKM, ALDOA, PGAM1, PKM2 and LDHA in OS. Enhanced glycolysis can promote enhanced metabolism of its branches (PPP and SSP), thereby promoting amino acid and lipid anabolism favorable to OS progression. The improvement of the fatty acid synthesis pathway and the MVA metabolism favor rapid growth and metastasis in OS. Expression of rate‐limiting enzymes in *β*‐oxidation of fatty acid is positive in approximately 90% of OS cases.^68^ The PPP is a primary source of NADPH, and SSP, glutamine metabolism, FAO, and the TCA cycle can also directly or indirectly provide NADPH for anabolic reactions and redox homeostasis offering reducing capacity.^139^ However, studies on PPP and lipid synthesis are inadequate, and studies related to fatty acid catabolism are scarce in OS. There are mtDNA mutations in OS, which may cause altered mitochondrial function. Mitochondrial dysfunction is present in glycolytic‐dominant OS cells, whether the increased reliance on glycolysis is due to inherent mitochondrial dysfunction or genetic alterations affecting the genes that maintain mitochondrial status. Whether OXPHOS‐dominated OS cells are less malignant than glycolysis‐dominated OS cells is due to metabolism influencing its malignancy or whether oncogenes contribute to tumor malignancy and metabolic alterations. It is unclear whether the abnormal metabolic pattern of tumors is a cause or a consequence of tumorigenesis, or both may be present, and considerable further research is required. Osteosarcoma is considered a disease caused by abnormal differentiation resulting from an abnormal interruption of the differentiation process of bone marrow mesenchymal stem cells to osteoblasts.^140^, ^141^, ^142^ In recent years, there has been growing evidence that the regulation of mitochondrial dynamics and function is critical for the successful differentiation of MSCs.^143^ Mitochondria are the site of OXPHOS in cells and are the hubs linking glucose, fatty acid and amino acid metabolism, and abnormalities in mitochondria can alter the metabolic pattern of cells. Osteogenic differentiation is a precisely regulated process. It is worth investigating whether abnormal disruption of the normal osteogenic differentiation process is associated with metabolic reprogramming and the role of metabolism on OS cell differentiation.

Shifts in tumor metabolism can be used as targets for cancer therapy. Relevant drugs targeting cancer metabolism are available, with most metabolic targets focused on cancer anabolism, such as the standard methotrexate (dihydrofolate reductase inhibitor) and 5‐fluorouracil (thymidylate synthase inhibitor).^144^ Current studies have found that some drugs can target OS metabolism and thus affect OS progression. For example, CDDO‐NFM (a triterpenoid) can inhibit OS cell growth by blocking the glycolytic process through the degradation of c‐MYC.^145^ The glycolysis inhibitor 2‐deoxy‐D‐glucose (2‐DG) can significantly delay metastasis and prolong survival in a model of OS after in situ surgery.^146^ Metformin increases the sensitivity of OS stem cells to cisplatin by inhibiting the expression of PKM2.^56^ LDHA inhibitors inhibit the proliferation of OS cells.^59^, ^60^ Ailanthone (AIL) inhibits OS cell proliferation, migration and invasion by downregulating SSP.^71^ The *α*‐linolenic acid inhibits the proliferation and invasion of OS cells by inhibiting FASN.^103^ Tumor cells are heterogeneous; different tumors or parts of the same tumor may have other metabolic profiles, and tumors can undergo metabolic switching to adapt to changes, making using a single metabolic inhibitor ineffective. The heterogeneity of tumor metabolism between tumor cells, microenvironment, and primary and metastatic sites is worth investigating. Osteosarcoma occurs during periods of vigorous growth when bone growth requires high energy, and the impact of targeted metabolic therapies on normal developmental processes needs to be considered.

## AUTHOR CONTRIBUTION

**Yulu Shi**: Conceptualization; writing; reviewing. **Xiaohan Yue**: Conceptualization. **Qing Luo**: Conceptulization; editing. All authors approved the final manuscript.

## CONFLICT OF INTEREST STATEMENT

Authors declare no conflict of interest.

## Data Availability

Data sharing not applicable to this article as no datasets were generated or analyzed during the current study.
